# Identification of tobacco leaf diseases using hyperspectral imaging and machine learning with SHAP interpretability analysis

**DOI:** 10.3389/fpls.2025.1711972

**Published:** 2026-01-06

**Authors:** Peng Luo, Yang Yang, Huilai Zhang, Man Yi, Xianguo Zhou, Yide Yang, Huabao Chen, Min Yan, Chunxian Jiang

**Affiliations:** 1 College of Agronomy, Sichuan Agricultural University, Chengdu, China; 2 Yibin Municipal Company of Sichuan Provincial Tobacco Corporation, Yibin, China

**Keywords:** hyperspectral imaging, machine learning, tobacco leaf diseases, disease classification, SHAP analysis

## Abstract

Tobacco leaf diseases significantly affect yield and quality, underscoring the need for rapid and non-destructive diagnostic tools. Although hyperspectral imaging (HSI) has been applied in tobacco pathology, most existing studies focus on single diseases and lack generalized, interpretable frameworks for multi-class identification. In this study, hyperspectral images of healthy leaves and four major diseases-brown spot, wildfire, Tobacco Mosaic Virus (TMV), and Potato virus Y (PVY)-were collected to construct a balanced, leaf-independent dataset. Pixels were grouped by leaf ID, and the entire dataset was strictly partitioned at the leaf level to prevent pixel-level data leakage and ensure generalization to unseen leaves. Multiple preprocessing techniques, wavelength-selection methods, and machine-learning classifiers were systematically compared. A compact ANN model integrating Savitzky-Golay preprocessing and SPA-based wavelength selection achieved the best overall performance while requiring only a small number of informative wavelengths. A Transformer model provided slightly stronger predictive capacity but depended on full-spectrum inputs and substantially higher computational cost. Pixel-level predictions enabled lesion-area-based severity estimation for the two leaf-spot diseases. SHAP analysis highlighted physiologically meaningful spectral regions associated with pigment absorption and structural variation. Overall, this study presents an efficient and interpretable HSI framework for multi-disease tobacco diagnosis, supporting the development of practical hyperspectral or multispectral systems.

## 1 Introduction

Tobacco is one of the cornerstone industries in China’s agricultural and rural economy (Fan, 2021). However, it is highly susceptible to several major foliar diseases, including brown spot, wildfire, Tobacco Mosaic Virus (TMV), and Potato virus Y (PVY), which can significantly reduce leaf yield and quality. These diseases differ in etiology and symptom expression. Their overlapping visual characteristics make accurate field identification challenging. Symptom variation among plant varieties, growth stages, and field environments further complicates diagnosis. Current diagnosis methods rely mainly on visual inspection and molecular assays. Visual inspection is fast and inexpensive but tends to be subjective and inconsistent, while molecular detection offers high specificity but requires destructive sampling and additional laboratory resources (Khakimov et al., 2022). These limitations highlight the need for non-destructive, objective approaches that can differentiate multiple disease types.

Hyperspectral imaging (HSI) has shown potential for plant disease detection because disease-induced changes in pigments, cellular structure, and water status can alter leaf reflectance across visible and near-infrared wavelengths (Sims and Gamon, 2002). By capturing continuous spectral information and providing spatial detail, HSI can reveal disease-related spectral patterns and lesion distribution (Cheshkova, 2022). Several studies have explored HSI for tobacco disease analysis. Dou et al. (2015) reported reflectance changes around 550 nm and red-edge shifts as disease severity increased. Zhu et al. (2017) achieved high TMV detection accuracy, and Gu et al. (2019) demonstrated early detection of Tomato Spotted Wilt Virus using hyperspectral measurements. However, these investigations mainly addressed single diseases or binary classification tasks, and therefore do not reflect multi-disease situations commonly encountered in cultivation.

Machine learning (ML) approaches such as SVM, RF, and neural networks have been widely used in hyperspectral classification of plant diseases across various crops (Golhani et al., 2018; Zhang et al., 2022a; Zhao et al., 2022a; Zhang et al., 2024a; Zhang et al., 2022b, Zhang et al., 2025). Transformer-based deep learning models have also shown promising performance in hyperspectral feature extraction due to their ability to model long-range spectral dependencies (He et al., 2021; Hong et al., 2022; Shu et al., 2024; Ullah et al., 2025). Despite these advances, their application to tobacco remains limited, and few tobacco studies have systematically compared ML and deep learning models under similar experimental conditions. Moreover, the performance of lightweight ML models combined with wavelength selection has not been fully examined relative to full-spectrum deep networks, particularly when dataset sizes are limited.

Another aspect that requires further attention is model interpretability. Many hyperspectral classification studies report high accuracy but provide limited information on how individual wavelengths contribute to model decisions. This limits the ability to connect spectral patterns with physiological processes. Explainable AI methods such as SHAP (Shapley, 1988; Lundberg and Lee, 2017) have been applied in several agricultural studies-for example, identifying important wavelengths for sweet potato quality prediction (Ahmed et al., 2025) and analyzing the contribution of environmental factors to crop yield (Wang et al., 2025b)-but have rarely been used in tobacco disease classification. Additionally, although lesion visualization has been explored in some contexts, the integration of multi-disease classification with pixel-level lesion mapping and severity estimation is still limited in tobacco research. These limitations show that, although substantial progress has been made, current studies still leave several important gaps unaddressed. Taken together, current HSI-based studies on tobacco have not yet fully addressed multi-disease conditions, model efficiency under limited data, or interpretable links between spectral features, spatial lesion patterns, and disease severity.

**Figure 1:**
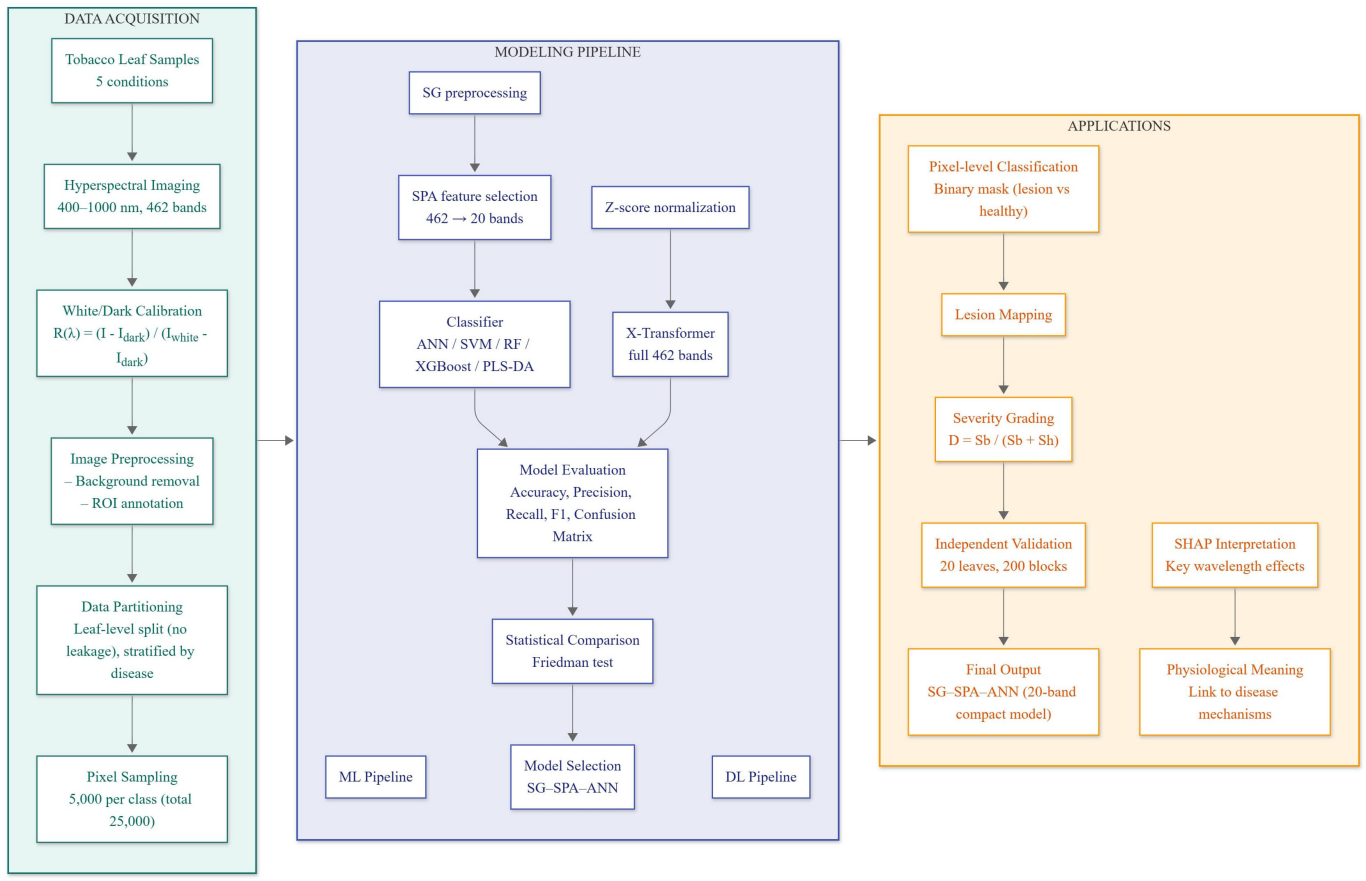
Workflow of the hyperspectral tobacco disease classification framework. The pipeline includes data acquisition and preprocessing, SG-based smoothing and SPA wavelength selection, model training using machine-learning and deep-learning classifiers, and downstream applications such as lesion mapping, severity estimation, and SHAP-based wavelength interpretation.

Given these observations, this study aims to develop a practical framework for classifying four major tobacco diseases-brown spot, wildfire, TMV, and PVY-along with healthy leaves using hyperspectral imaging. We systematically evaluated multiple preprocessing methods, wavelength-selection algorithms, and ML classifiers to identify combinations suitable for multi-disease classification. A Transformer-based deep learning model was included as a benchmark to compare full-spectrum performance with feature-selected ML models. To improve interpretability, SHAP was applied to analyze the contribution of key wavelengths and relate them to known spectral-physiological responses. Furthermore, pixel-level predictions were used to visualize lesion distribution and estimate severity for brown spot and wildfire. The workflow of the proposed framework is summarized in **Figure 1** and is intended to provide a basis for developing more interpretable and computationally efficient methods for tobacco disease identification under controlled experimental conditions.

## 2 Materials and methods

### 2.1 Plant materials

The trial utilized the tobacco cultivar Yunyan 99, grown in two field plots in Xianfeng Miao Ethnic Township, Yibin City, Sichuan Province, China. The region has an average annual temperature of 16-18°C, uniform water and fertilizer management were applied in experimental areas to minimize cultivation-related variation. In July 2025, leaves at an intermediate developmental stage between vigorous growth and maturity were collected from mature fields. A total of 95 leaves were collected, with the following distribution among disease categories: brown Spot (35 leaves), wildfire (33 leaves), PVY (14 leaves), TMV (8 leaves), and healthy (5 leaves). The relatively small numbers of TMV, PVY, and healthy leaves reflect the low field incidence of these categories during the sampling period rather than intentional undersampling, but they also limit the environmental and genetic diversity represented in dataset and are therefore acknowledged as a limitation of this study. All leaves were randomly collected from individual plants in the two fields, immediately sealed in bags, and transported to the laboratory to preserve sample integrity. Three tobacco experts conducted visual assessments of the leaves, strictly following GB/T 23222–2008 Survey Method for Classification of Tobacco Diseases and Pests. The disease-severity grading criteria defined in this national standard, including lesion-area thresholds, are summarized in **Table 1** and were used to determine disease severity based on leaf symptoms (e.g., spot morphology, color changes).annual precipitation of about 1,500 mm, and average relative humidity of 85-90%. Uniform water and fertilizer management and integrated pest control measures were applied across all experiment.

**Table 1:** Disease severity grading of tobacco brown spot and wildfire diseases.

Grade	Proportion of lesion area on the leaf surface
Level 0	No lesions on the whole leaf
Level 1	Lesion area accounts for less than 1% of leaf area
Level 3	Lesion area accounts for 2%-5% of leaf area
Level 5	Lesion area accounts for 6%-10% of leaf area
Level 7	Lesion area accounts for 11%-20% of leaf area
Level 9	Lesion area accounts for more than 21% of leaf area

### 2.2 Hyperspectral imaging data acquisition

**Figure 2:**
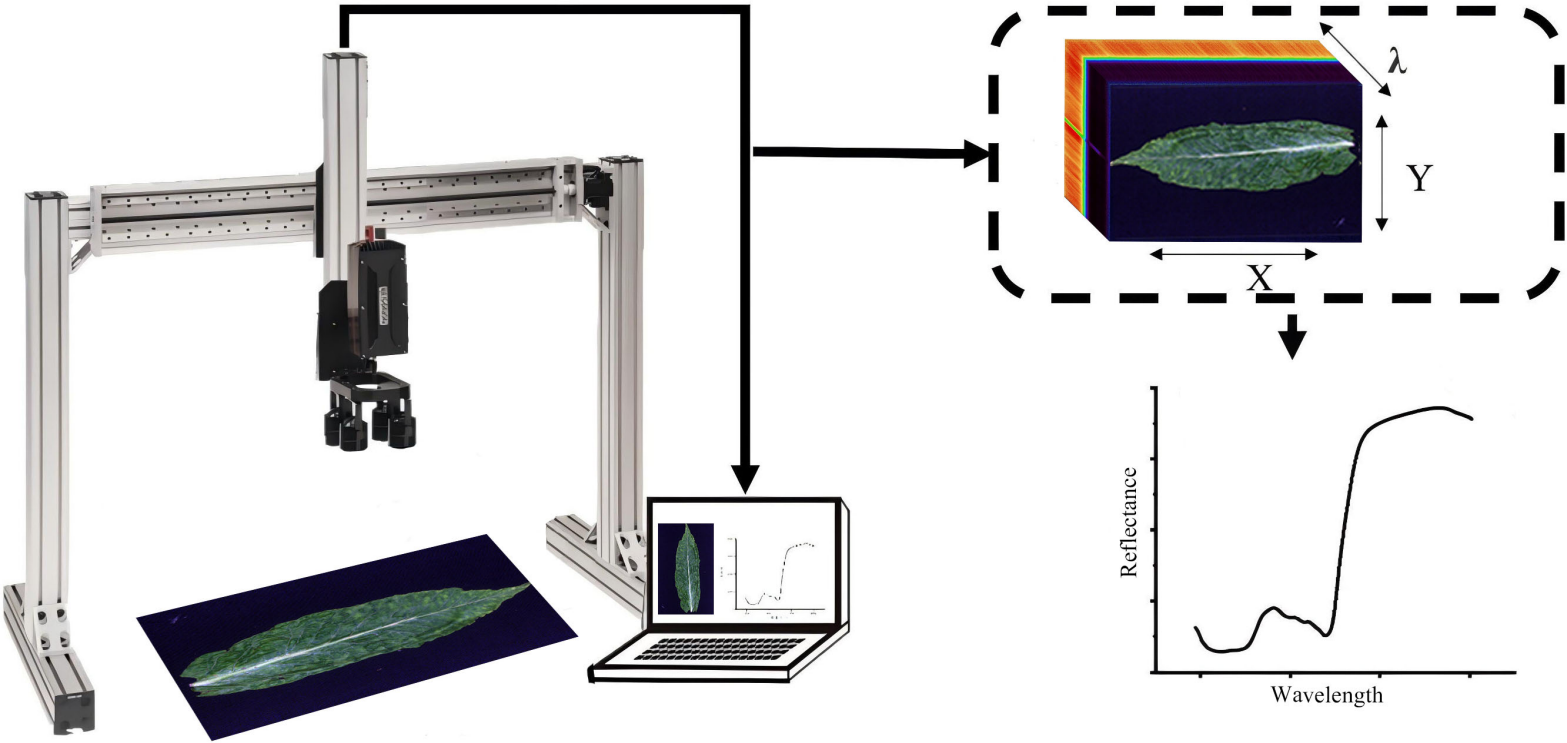
Hyperspectral imaging system used in this study. A push-broom hyperspectral camera was mounted on a custom gantry above the sample platform. A tobacco leaf was placed on the platform, and image acquisition was controlled through a computer. The camera produced a three-dimensional hyperspectral cube with spatial dimensions X and Y and a spectral dimension λ, and a reflectance spectrum could be extracted from each pixel.

In this study, hyperspectral data were acquired using a Pika XC2 hyperspectral imager (Resonon Inc., USA). **Figure 2** provides an overview of the hyperspectral imaging system used in this study, including the custom-built gantry. Its spectral range was 400–1000 nm, with a spatial resolution of 1600 pixels and a spectral resolution of approximately 1.9 nm, providing a total of 462 spectral bands with a spectral sampling interval of about 1.3 nm. The hyperspectral imager was mounted on this gantry, with a black non-woven fabric used as the background. The hyperspectral camera, linear moving platform, computer, and light source were connected and powered, with the vertical distance between the camera and the platform maintained at approximately 20 cm. The illumination was provided by four 150 W halogen lamps, which were preheated for 20 minutes to ensure output stability. The lamps were arranged symmetrically at an approximate 45° angle to the sample surface to achieve uniform, diffuse lighting and minimize specular reflection. After loading the equipment onto the computer, the platform was adjusted to the initial scanning position, and a standard whiteboard was placed for auto-exposure, focusing, dark current acquisition, and whiteboard calibration. The image aspect ratio was adjusted according to the size of each tobacco leaf. The image width was fixed at 1600 pixels. The scan height was adjusted automatically according to each leaf’s length. In our dataset, the scan height typically ranged from 800 to 1500 pixels. The aperture was set to 2.8, the camera frame rate to 20 Hz, the camera height to approximately 35 cm, the camera gain to 5, the exposure time to about 47 ms, and the linear platform moving speed to 0.132 cm/s. All image acquisitions were conducted in a darkroom to ensure that the measurements were not affected by fluctuating ambient light. After completing the acquisition of hyperspectral reflectance data for each sample, or after adjusting the platform speed or exposure time, standard whiteboard calibration was required to maintain consistency. The hyperspectral reflectance of each tobacco leaf sample was calculated according to Equation 1:

(1) $$\begin{array}{c} R=\frac{I_{raw}-I_{dark}}{I_{white}-I_{dark}} \end{array}$$

In equation, $I_{raw}$ represents the unprocessed Digital Number (DN) values, indicating the relative light intensity recorded by the sensor; $I_{white}$ represents the white reference data, obtained from a standard white reference panel and representing full reflectance; $I_{dark}$ represents the dark current data, collected by covering the lens or closing the shutter and representing the sensor noise signal; and R represents the calibrated hyperspectral reflectance value of the tobacco leaf sample, reflecting the true spectral characteristics.

### 2.3 Background removal for hyperspectral images

**Figure 3:**
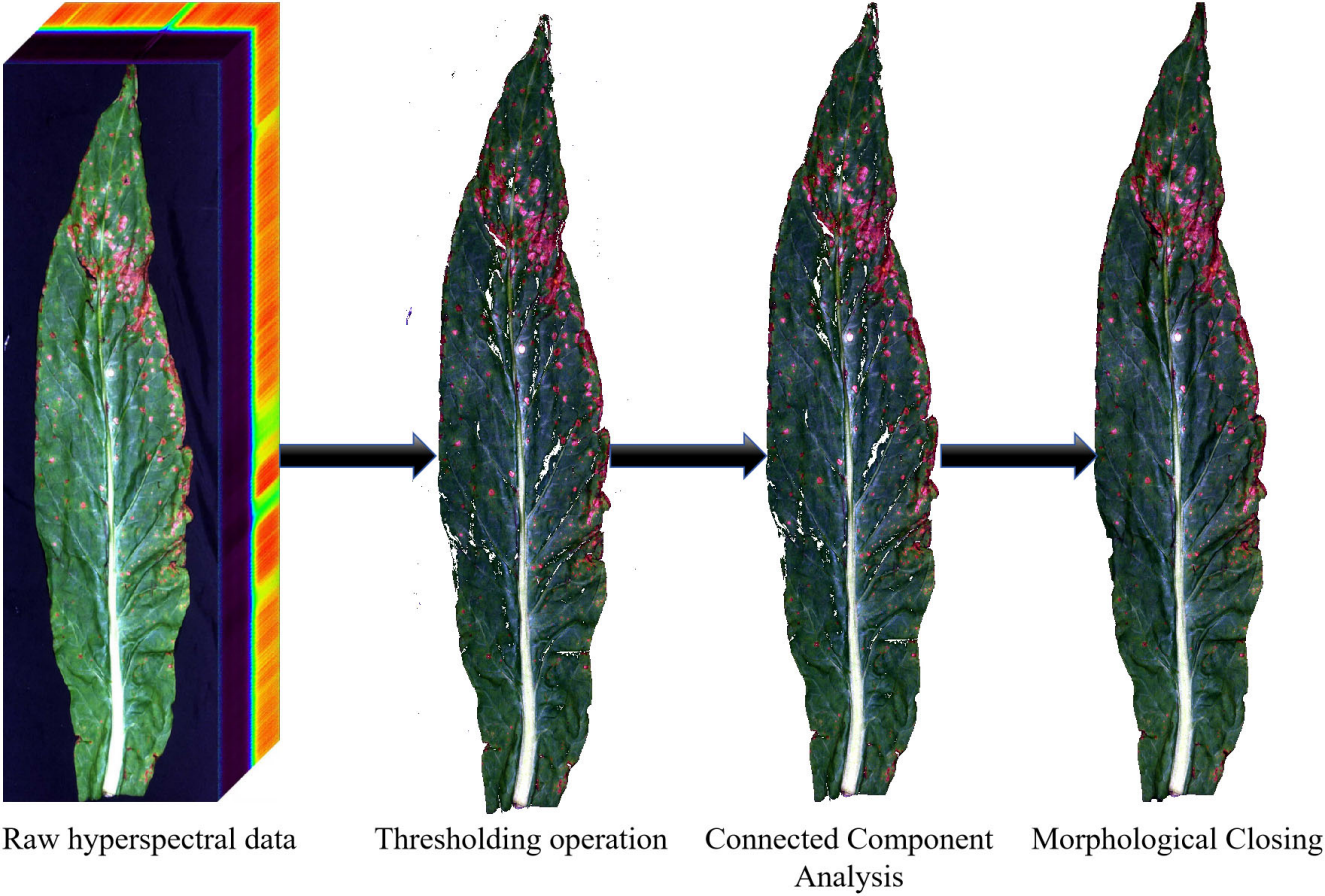
Background removal workflow for hyperspectral tobacco leaf images. The procedure included grayscale thresholding, connected component analysis, and morphological closing to extract the leaf region from the background.

The three-step background removal workflow for hyperspectral tobacco leaf images is shown in **Figure 3**. The workflow included thresholding, connected component analysis, and morphological closing. This processing was implemented in Python using the OpenCV package. The 163rd band with a center wavelength of 563.33 nm was selected for initial segmentation because it provided clear contrast between the leaf and the background. The grayscale range of 200 to 700 was determined after examining representative images. The black non-woven background consistently appeared below 200. All leaf tissues, including both healthy and diseased regions, appeared between 200 and 700. Values above 700 were removed because they did not contain valid leaf information. Because a uniform black non-woven fabric was used as the background, the same threshold was applied to all samples. Connected component analysis was used to remove regions outside the leaf. The pixel area of each connected region was calculated, and the largest region was kept. Finally, a morphological closing operation was applied to the retained leaf region to fill small gaps and smooth the boundary of the leaf (Sreedhar and Panlal, 2012).

### 2.4 Data annotation and spectral preprocessing

After hyperspectral image acquisition, each hyperspectral image cube was processed independently and corresponded to one tobacco leaf. ROIs were manually delineated in ENVI 5.6. For each disease class, ROIs were selected from multiple leaves. The selected regions included lesion centers, lesion margins, transition zones, and nearby healthy tissues. Pixel spectra exported from ENVI inherently retained their leaf origin because each cube corresponded to one leaf. The dataset was divided into training, validation, and test sets at the leaf level before pixel sampling. All pixels from each leaf were kept within the same subset to avoid data leakage. Within each subset, pixels from leaves belonging to the same disease class were pooled. From each disease class, 5,000 spectra were randomly selected by stratified sampling. This yielded 25,000 spectra (5,000 per class) and a balanced dataset. To improve data quality and classification performance (Yan, 2025), we compared four preprocessing methods for the machine-learning models, namely SG (Luo et al., 2005) smoothing, SNV (Vu et al., 2018) transformation, MMS (Fan et al., 2024), and SS (de Amorim et al., 2023). In the deep learning experiments, we applied Z-score normalization computed from the training set only. The training-set mean and standard deviation were used to scale the validation and test sets.

### 2.5 Feature wavelength selection algorithms

However, the presence of wavelength redundancy and noise in hyperspectral data may increase computational cost and degrade prediction performance (Morales et al., 2021; Elmaizi et al., 2022; Zhao et al., 2022b). To enhance training efficiency and improve classification accuracy, three typical feature wavelength selection methods were evaluated, namely Competitive Adaptive Reweighted Sampling (CARS) (Li et al., 2009), Successive Projections Algorithm (SPA) and Least Angle Regression (LARS). Each method is based on a distinct selection strategy. CARS iteratively updates the weights of individual wavelengths and selects the most discriminative subset across the full spectrum. Based on a PLS model, the importance of wavelengths is evaluated by Root Mean Square Error of Cross-Validation (RMSECV), and the most important wavelengths are extracted. SPA (Soares et al., 2013) reduces the multicollinearity among variables by selecting wavelengths sequentially that maximize the variance explanation while preserving mutual independence. LARS (Efron et al., 2004) selects wavelengths that are most correlated with the target variable and then updates the regression coefficients to incrementally build the predictive model.

### 2.6 Classification models for tobacco leaf diseases

#### 2.6.1 Computational environment

The experiments in this study were conducted using the following hardware and software environment to maintain consistency in the experimental setup. The hardware setup included an NVIDIA GeForce RTX 4080 GPU with 16 GB GDDR6X memory, utilizing CUDA 11.8 for GPU acceleration. The software environment consisted of Python 3.8, PyTorch 2.7.1, CUDA 11.8, and scikit-learn 1.6.1 for machine learning tasks. A fixed random seed of 42 was applied across all machine learning and deep learning models to standardize model initialization.

#### 2.6.2 Machine learning models

In this study, we investigated the performance of several commonly used machine learning algorithms for hyperspectral classification of tobacco leaf diseases. We compared five representative models, including artificial neural networks (ANN) (Golhani et al., 2018), extreme gradient boosting (XGBoost) (Chen and Guestrin, 2016), random forests (RF) (Wójtowicz et al., 2021), support vector machines (SVM) (Zhang et al., 2022a; Ray et al., 2025), and partial least squares discriminant analysis (PLS-DA) (Zhang et al., 2024b). These models represent a wide range of modeling approaches, from traditional statistical models to ensemble-based models, and were selected to represent commonly used strategies for spectral-data classification. Each model was trained independently on the same data, to compare performance in terms of classification accuracy, robustness to variations in input data, and generalization to previously unseen data.

#### 2.6.3 X-transformer model: architecture and training

In addition to these conventional models, we also evaluated a Transformer-based model to provide a deep-learning reference model. Specifically, we used a Transformer implemented in the x-transformers open-source library (Wang, 2025a) and compared its performance with that of traditional machine learning models. The Transformer model uses self-attention to model the relationships between different spectral bands and thus captures global dependencies in the input sequence (Vaswani et al., 2017). By using multi-head attention, the model can learn multiple spectral features in parallel from different representation subspaces. To preserve the sequential information of the hyperspectral data, learned positional encodings were used to provide band-order information. These design features make the Transformer an effective model for spectral classification tasks (Hong et al., 2022).

The X-Transformer model used in this study takes the full vector of 462 hyperspectral reflectance bands as input. Since the raw spectral signals are not directly suitable for deep learning, we linearly projected them into a 256-dimensional embedding vector. To encode the positional information of each spectral band, we employed learned positional embeddings optimized jointly with the model during training, rather than using fixed sinusoidal encodings. The model architecture consists of six stacked encoder layers, each equipped with eight attention heads. A dropout rate of 0.1 was applied to both the attention and feedforward layers as a standard configuration. After each attention block, a feedforward network with an intermediate dimension of 1,024 was applied as part of the model architecture. To aggregate the spectral information across all bands, we applied average pooling over the sequence dimension, thereby transforming the band-wise representations into a global spectral feature vector. This pooled representation was then fed into a linear classification layer to produce the final disease category predictions. In total, the model contains approximately 4.84 million parameters.

For disease identification, the model outputs five classes corresponding to the five major tobacco leaf diseases. The training dataset comprised 25,001 samples, which were stratified and split into a training set of 17,500 samples (70%) and a validation set of 7,501 samples (30%) to retain class proportions across subsets. We used the AdamW optimizer with an initial learning rate of 3 × 10–^4^ and a weight decay of 1 × 10–^4^ to introduce additional L2 regularization on the model parameters. The batch size was set to 64 for efficient gradient computation. To accelerate convergence, we used the ReduceLROnPlateau scheduler, which automatically decreases the learning rate by a factor of 0.5 when the validation loss stops decreasing for five consecutive epochs, with a minimum learning rate threshold of 1 × 10^-6^. Additionally, we adopted an early-stopping criterion with a patience of 10 epochs as part of the training procedure. Before training, we applied Z-score normalization to standardize the input data, mitigating the impact of differing feature scales. The normalization statistics (mean and standard deviation) were computed exclusively from the training set to prevent data leakage across subsets.

### 2.7 Data partitioning and model evaluation

Since we aimed to keep the class proportions balanced during the data partitioning process, we split the data into a 70% training set and a 30% test set. A sample represents a pixel-wise spectral signature. To obtain more stable performance estimates, we used a 5-fold stratified cross validation scheme during training (Lumumba et al., 2024). For model evaluation, we used common evaluation metrics such as accuracy, precision, recall, and the F1-score, and summarized the model output using a confusion matrix to display class-wise performance. Additionally, a confusion matrix was generated to visualize the class-wise classification performance (Rainio et al., 2024).

The calculation formulas for Accuracy, Precision, Recall, and F1-Score are as follows (Equations 2–5).

(2) $$\begin{array}{c} \text{Accuracy}=\frac{TP+TN}{TP+TN+FP+FN} \end{array}$$

(3) $$\mathrm{Pr}ecision=\frac{TP}{TP+FP}$$

(4) Recall=TPTP+FN

(5) F1-Score=2×Recall×PrecisionRecall+Precision

*TP* represents true positives, which are samples that belong to the positive class and are predicted as positive. *FN* corresponds to false negatives, which are samples that were actually positive but are incorrectly classified as negative. In contrast, *FP* refers to false positives, which occur when negative samples were incorrectly predicted as positive by the model. *TN* represents true negatives, indicating samples that truly belong to the negative class and were correctly predicted as negative.

### 2.8 Model interpretation based on SHAP

In order to interpret how different spectral wavelengths contribute to the model predictions, we used the SHAP (Shapley Additive exPlanations) algorithm as an interpretability method. SHAP is a game theory method that uses Shapley values to provide a fair way to measure the contribution of each feature by averaging its marginal effect over all possible coalitions of input features (Lundberg and Lee, 2017). For the feature-importance analysis, SHAP values were computed for all test samples using a KernelExplainer applied to the trained ANN model. The SHAP values formed a three-dimensional array with the number of samples, the number of features, and the number of disease classes. The absolute SHAP values were averaged across all test samples and all disease classes to obtain one global importance value for each feature. SHAP values were computed using a single ANN model trained on the full training set, without averaging across cross-validation folds. In this work, SHAP values were used to quantify the impact of different spectral wavelength features on the classification of tobacco leaf diseases.

The SHAP value can be computed as below (Equation 6):

(6) $$f(x)=\phi_{0}+\sum_{i=1}^{M}\phi_{i}$$

Here, *f(x)* is the prediction of the model, *M* is the total number of features, $\left(\phi_{0}\right)$ represents the baseline prediction (i.e., the mean output of all samples in the training set), and $\left(\phi_{\text{i}}\right)$ represents the SHAP value of the (i) feature, indicating its contribution to the current prediction. If $(\phi_{\text{i}}>0)$, the feature has a positive effect on the prediction result; if $(\phi_{\text{i}}<0)$, it has a negative effect.

To reveal the overall influence patterns and importance ranking of spectral features, this study generated two SHAP visualization outputs: (1) SHAP feature importance bar charts, displaying the average absolute SHAP values for each disease and feature, sorted in descending order of importance. This visually reflects the overall contribution of different spectral bands to model outputs (Ponce-Bobadilla et al., 2024); (2) SHAP bee swarm plot for each disease, in which the SHAP values of all samples are plotted by feature, simultaneously showing feature importance, the relationship between feature values and the sign of SHAP values, as well as the concentration and dispersion trends of feature effects, thus providing a comprehensive evaluation of the role of spectral features in classification decisions (Afroj et al., 2025; Contreras et al., 2024). For both types of SHAP visualizations, the top 20 spectral wavelengths ranked by mean absolute SHAP value were selected to focus on the most influential bands and improve interpretability.

### 2.9 Disease severity grading of brown spot and wildfire

According to the severity criteria defined in **Table 1**, derived from GB/T 23222-2008, the severity level of tobacco brown spot and wildfire is determined based on lesion area (Xu et al., 2024). In this study, the trained classification model was applied to hyperspectral images of these two diseases, to generate pixel-level differentiation between healthy tissues and lesion areas. The prediction results produced a binary classification mask, where each pixel label represents its corresponding category. To visualize the lesion regions, the mask image was pseudo-colored, with healthy regions labeled in green and lesion regions labeled in red. Based on this result, the lesion area ratio (D) was calculated using Equation 7:

(7) $$D=\frac{S_{b}}{S_{b}+S_{h}}\times 100\%$$

Where *D* represents the lesion area ratio, $S_{b}$ represents the total number of pixels identified as lesions, and $S_{h}$ represents the total number of pixels identified as healthy tissues.

In this study, 10 independent tobacco leaves were selected for each disease type, giving a total of 20 leaves. Each leaf was divided into 10 small blocks, yielding 200 blocks for severity grading evaluation. These independent leaves were not included in the training set. Based on the calculated lesion area ratio, the severity level was assigned according to **Table 1**. To assess model performance, a confusion matrix was constructed to compare the model predictions with the grades assigned by human experts.

## 3 Results

### 3.1 Spectral characteristics of different diseases

**Figure 4:**
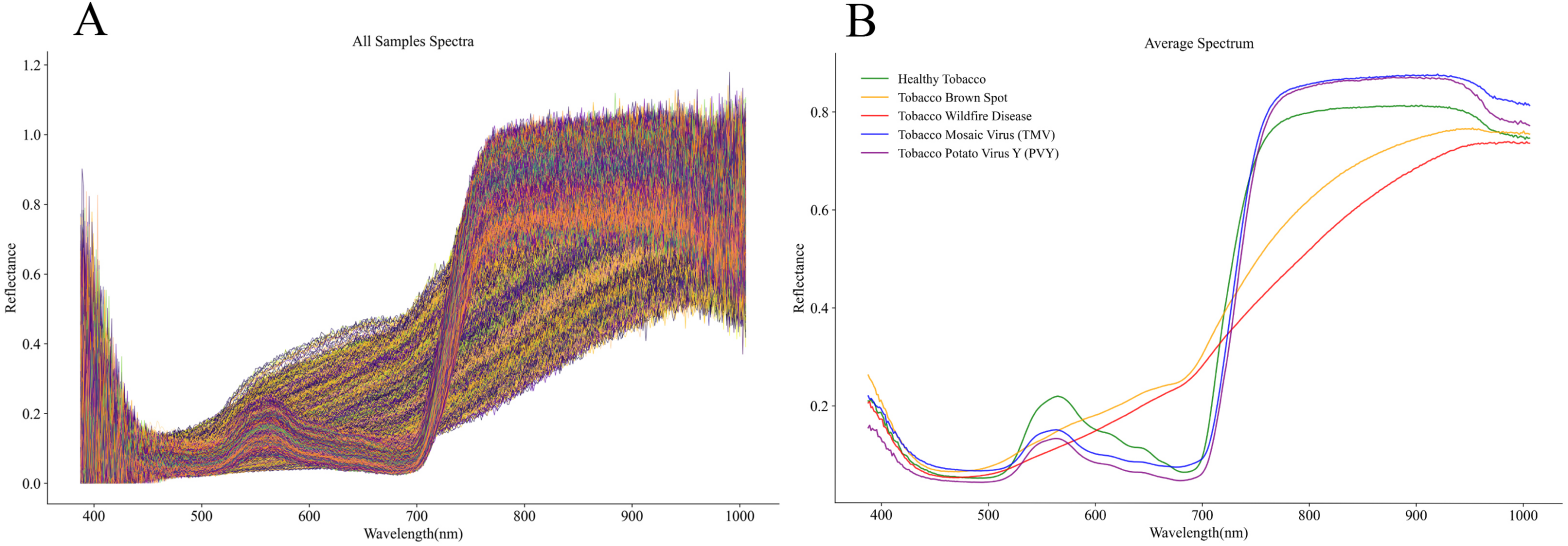
Original and average spectral curves of different tobacco diseases. **(A)** Spectral curves of all samples; **(B)** average spectral curves.

By performing spectral extraction on representative ROIs within tobacco leaves affected by different diseases, we obtained both the raw spectral reflectance curves (**Figure 4A**) and the mean spectral reflectance curves (**Figure 4B**) for each disease type. Analysis revealed significant differences in spectral characteristics across visible-near infrared wavelengths (400–1000 nm) among the different diseases. In the average spectral curves (**Figure 4B**), the reflectance of brown spot and wildfire was generally lower across the entire band range than that of healthy tobacco, TMV, and PVY, and no distinct reflection peak appeared for the two diseases in the 500–600 nm range. In the near-infrared region of 700–1000 nm, the reflectance of brown spot and wildfire remained significantly lower than that of healthy samples. In contrast, the reflectance of brown spot remained slightly higher than that of wildfire at all bands. The spectral curves of TMV and PVY were relatively close to those of healthy, but the reflectance at the green peak around 550 nm was noticeably lower; in the near-infrared region of 700–1000 nm, however, it became higher than that of healthy. These spectral characteristics differed from those of healthy tobacco in both overall trends and specific wavelength regions.

### 3.2 Spectral data preprocessing

In order to prepare the spectral data and highlight wavelength-dependent variation, this study selected four methods of SG, SNV, MMS, and SS to preprocess the raw spectra of tobacco leaves. The results are shown in.

**Figure 5:**
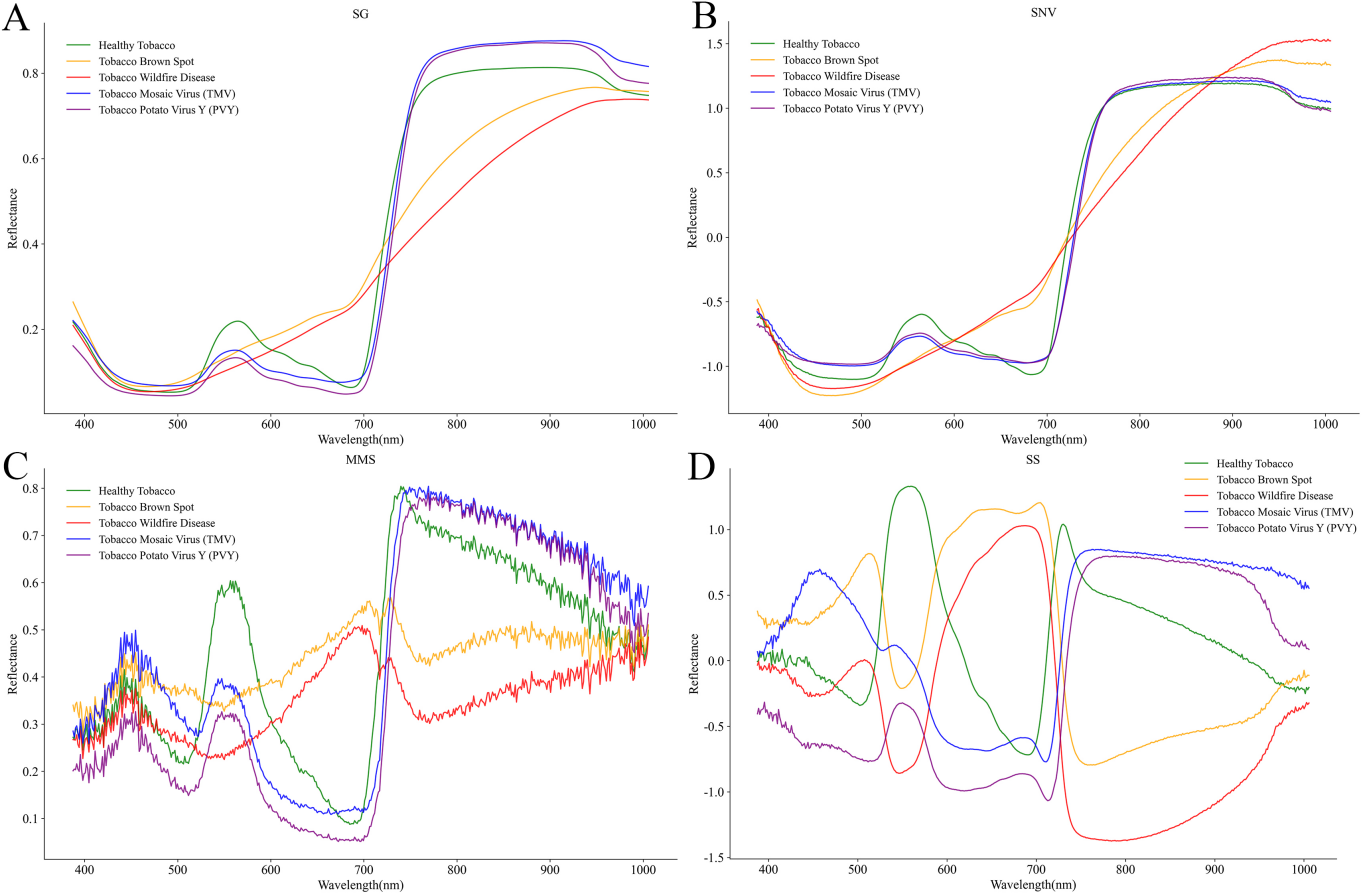
Spectral data preprocessing of tobacco samples. **(A)** SG; **(B)** SNV; **(C)** MMS **(D)** SS.

**Figure 5A**: the SG filter suppressed high-frequency noise and produced smoother spectral curves. After SNV preprocessing, reflectance differences among samples became more distinct in the 400–500 nm range (**Figure 5B**), and the spectral distribution became more compact in the 500–700 nm range. When MMS normalization was used, the reflectance difference among samples became more distinct in 600–800 nm range, and the disease influence on the leaf spectra became more distinct (**Figure 5C**). After SS preprocessing, overall changes in reflectance became more distinguishable, particularly within the 400–800 nm range, where spectral variation trends across samples were further emphasized (**Figure 5D**). It should be noted that compared with SG, which had a noise-suppressing effect, the increased fluctuations and saw-tooth patterns observed in **Figures 5B–D** were not caused by additional noise. Instead, these fluctuations arose from the amplification of subtle reflectance variations present in the raw spectra, reflecting underlying wavelength-dependent differences among disease types.

### 3.3 Full-spectrum classification under different preprocessing methods

This study investigated the impact of different spectral preprocessing techniques on the performance of classification models based on full-spectrum reflectance data. As shown in **Table 2**, four preprocessing methods (SG, MMS, SS, and SNV) were evaluated in combination with five classifiers. To statistically compare the 20 model configurations, a Friedman non-parametric test was applied to the sample-level prediction results. The test indicated significant performance differences among models (χ² = 12541.19, p< 0.0001), and the corresponding average ranks are reported in **Table 2**.

**Table 2:** Classification models based on full-spectral data with diverse preprocessing methods.

Preprocessing	Model	Dataset type	Accuracy	Precision	Recall	F1-score	CV accuracy	Avg. rank
SG	ANN	Train	99.64%	99.64%	99.64%	99.64%	99.43 ± 0.14%	9.86
		Test	98.93%	98.94%	98.93%	98.93%		
	SVM	Train	100.00%	100.00%	100.00%	100.00%	99.37 ± 0.32%	9.98
		Test	97.90%	97.93%	97.90%	97.90%		
	RF	Train	83.56%	85.06%	83.56%	83.29%	83.29 ± 0.20%	11.44
		Test	82.67%	84.45%	82.67%	82.42%		
	PLS_DA	Train	88.10%	87.99%	88.10%	87.98%	88.21 ± 0.73%	10.98
		Test	88.47%	88.43%	88.47%	88.36%		
	XGBoost	Train	99.44%	99.45%	99.44%	99.44%	98.86 ± 0.29%	9.99
		Test	97.50%	97.53%	97.50%	97.50%		
MMS	ANN	Train	99.74%	99.74%	99.74%	99.74%	99.44 ± 0.21%	9.87
		Test	98.73%	98.74%	98.73%	98.73%		
	SVM	Train	98.99%	99.01%	98.99%	98.99%	98.80 ± 0.23%	9.90
		Test	98.37%	98.37%	98.37%	98.37%		
	RF	Train	82.21%	84.07%	82.21%	81.84%	81.97 ± 0.70%	10.02
		Test	81.40%	83.70%	81.40%	81.01%		
	PLS_DA	Train	85.94%	86.03%	85.94%	85.80%	86.01 ± 0.71%	11.73
		Test	86.17%	86.29%	86.17%	86.02%		
	XGBoost	Train	99.60%	99.60%	99.60%	99.60%	98.66 ± 0.26%	10.10
		Test	96.47%	96.55%	96.47%	96.46%		
SS	ANN	Train	99.20%	99.74%	99.74%	99.74%	99.05 ± 0.32%	9.90
		Test	98.73%	98.74%	98.73%	98.73%		
	SVM	Train	99.90%	99.90%	99.90%	99.90%	97.81± 0.14%	10.02
		Test	97.47%	97.47%	97.47%	97.47%		
	RF	Train	81.81%	83.88%	81.81%	81.43%	81.57 ± 0.73%	11.53
		Test	80.89%	82.94%	80.89%	80.52%		
	PLS_DA	Train	86.04%	86.13%	86.04%	85.89%	78.73 ± 0.71%	11.25
		Test	85.63%	85.63%	85.63%	85.45%		
	XGBoost	Train	98.83%	98.86%	98.83%	98.83%	97.10 ± 0.24%	10.48
		Test	97.16%	97.16%	97.11%	97.10%		
SNV	ANN	Train	99.54%	99.54%	99.54%	99.54%	98.53 ± 0.33%	10.01
		Test	96.17%	96.16%	96.17%	96.16%		
	SVM	Train	98.60%	98.61%	98.60%	98.60%	98.24 ± 0.32%	10.07
		Test	97.40%	97.40%	97.40%	97.40%		
	RF	Train	89.56%	89.79%	89.56%	89.53%	89.53 ± 0.90%	10.90
		Test	89.47%	89.72%	89.47%	89.43%		
	PLS_DA	Train	91.14%	91.46%	91.14%	91.09%	91.04 ± 0.46%	10.74
		Test	90.80%	91.10%	90.80%	90.75%		
	XGBoost	Train	99.63%	99.63%	99.63%	99.63%	98.68 ± 0.41%	10.12
		Test	96.47%	96.47%	96.47%	96.47%		

For the ANN model, SG preprocessing yielded a test accuracy of 98.93%, a training-test gap of 0.71 percentage points, and a 5-fold cross-validation accuracy of 99.43 ± 0.14%. In comparison, the test accuracy of SNV for ANN was 96.17%, with a cross-validation accuracy of 98.53 ± 0.33%. The difference in test accuracy between SG and SNV (approximately 2.76 percentage points) exceeded the variability observed in cross-validation. For nonlinear models such as ANN, SVM, and XGBoost, the test accuracies under SG, MMS, and SS were generally close, with ANN showing very similar results for all three preprocessors (98.93%, 98.73%, and 98.73%, respectively). For SVM, SG and MMS yielded test accuracies about 0.5-1.0 percentage points higher than SS and SNV, while for XGBoost the SG-preprocessed model achieved the highest test accuracy (97.50%), followed by SS (97.16%), with MMS and SNV both at 96.47%, although the absolute differences were modest.

In contrast, for RF and PLS-DA, SNV produced higher accuracies than SG, MMS, and SS (e.g., 89.47% vs. 82.67%, 81.40%, and 80.89% for RF; 90.80% vs. 88.47%, 86.17%, and 85.63% for PLS-DA.) and, in terms of average rank, the MMS configuration achieved the best rank for RF (10.02), whereas SNV achieved the best rank for PLS-DA (10.74). Overall, SG, MMS, and SS yielded similar accuracies for ANN, SVM, and XGBoost, whereas SNV was associated with higher performance for RF and PLS-DA. This pattern is consistent with the denoising effect of SG-based smoothing for flexible nonlinear classifiers and with the ability of SNV to reduce multiplicative intensity and scatter effects that influence tree-based and projection-based models. In subsequent analyses, SG was selected as the primary preprocessing method.

### 3.4 Classification models based on characteristic bands

#### 3.4.1 Characteristic bands selection

**Figure 6:**
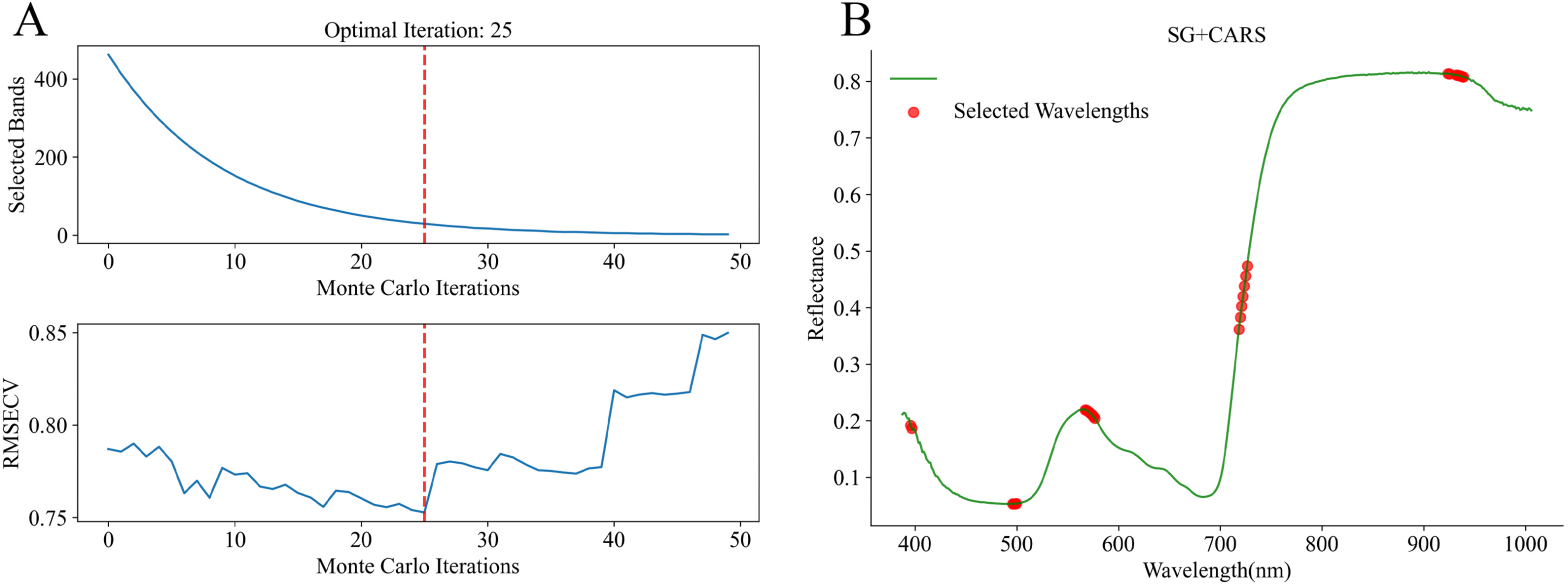
Process and result of characteristic band selection using CARS. **(A)** variation of selected bands and RMSECV with Monte Carlo iterations; **(B)** characteristic bands selection result.

In this study, after SG preprocessing, three algorithms, CARS, SPA, and LARS, were used for characteristic bands selection. The results of CARS are presented in **Figure 6**. With the increase in the number of Monte Carlo iterations, the number of selected bands gradually decreased and tended to stabilize, while the RMSECV showed a trend of first decreasing and then increasing (**Figure 6A**). At the 25th iteration, the RMSECV reached its minimum value, and the number of retained bands decreased to 29 (**Figure 6B**). The results of SPA are presented in.

**Figure 7:**
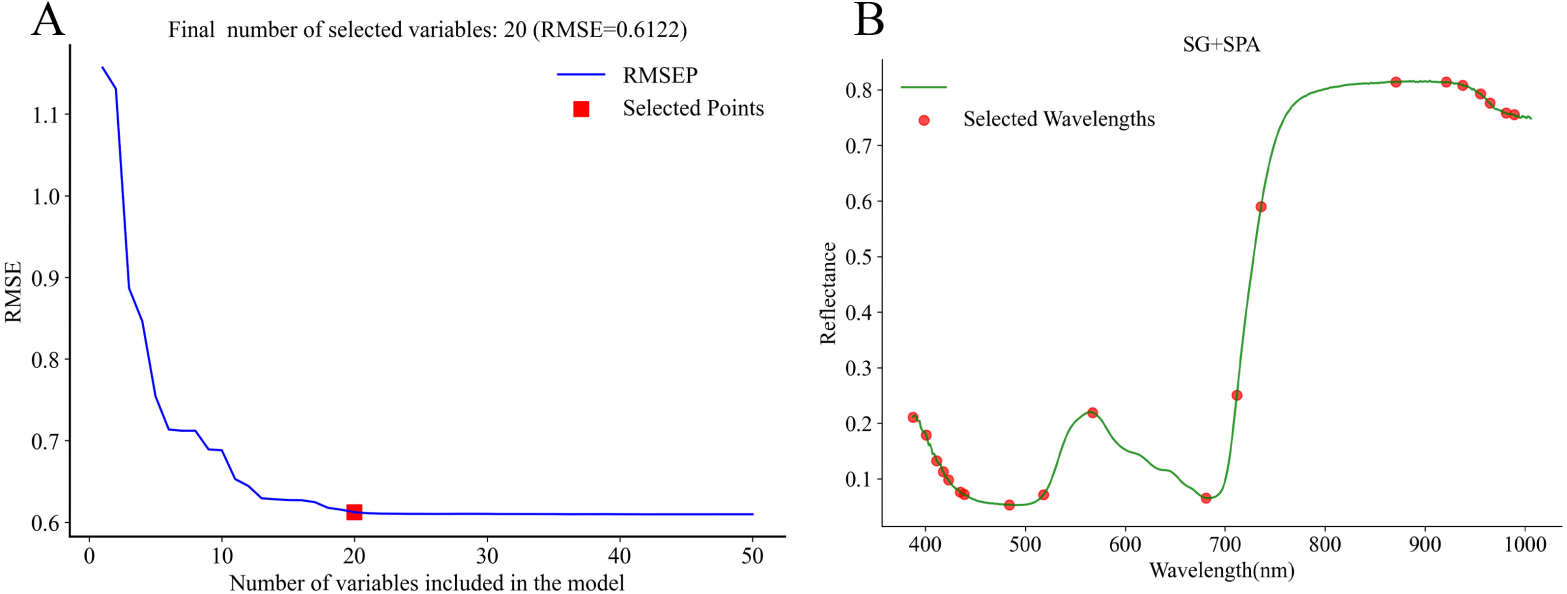
RMSEP curve and the result of characteristic bands selection of SPA. **(A)** RMSEP curve; **(B)** characteristic bands selection result.

**Figure 8:**
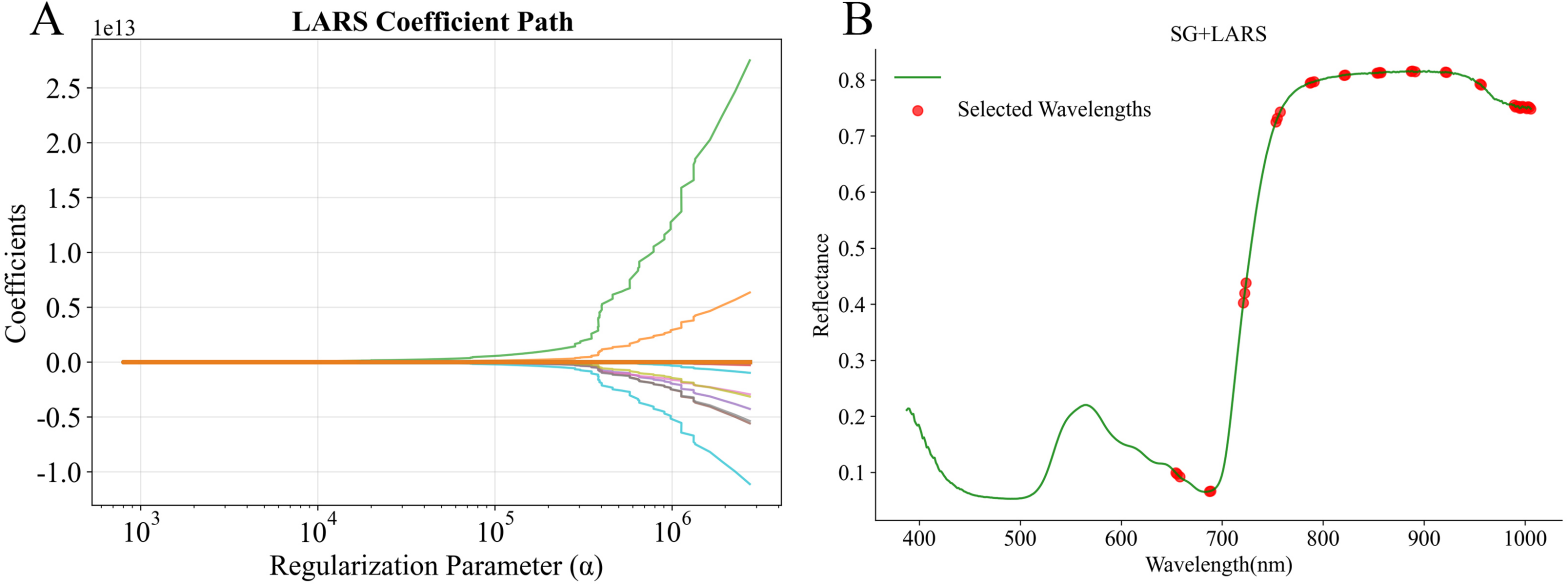
LARS coefficient path plot and results of characteristic bands selection. **(A)** LARS coefficient path plot; **(B)** characteristic bands selection result.

**Figure 7**. SPA, based on a forward selection strategy and vector projection analysis, gradually extracted feature subsets from the full spectrum using a combination of relevance assessment and redundancy control. As the number of selected bands increased, the prediction error (RMSEP) steadily decreased, ultimately stabilized at RMSEP = 0.6122 using 20 feature variables. The results of LARS are presented in **Figure 8**. This method followed a stepwise regression strategy in which variables were introduced according to their correlation with the dependent variable. In total, 40 bands were retained. LARS retained a larger number of spectral bands compared with CARS and SPA.

#### 3.4.2 Construction and analysis of classification models

##### 3.4.2.1 Traditional machine learning models

**Table 3** summarizes the performance of different combinations of SG-based feature selection methods (SPA, LARS, CARS) and classifiers (ANN, XGBoost, SVM, PLS-DA, RF). To provide an objective comparison among the 15 feature-band models, a Friedman non-parametric test was conducted on the sample-level prediction results. The test indicated statistically significant performance differences (χ² = 9347.20, p< 0.0001), and the corresponding average ranks for each model are reported in **Table 3**. Among the evaluated configurations, SG-SPA-ANN achieved the lowest average rank (7.34) in the Friedman test, with training and test accuracies of 99.33% and 98.88%, and a 5-fold cross-validation accuracy of 99.20 ± 0.14%. XGBoost combined with SG-SPA yielded a test accuracy of 97.01% and a cross-validation accuracy of 97.57 ± 0.29%, with an average rank of 7.45. However, the SG-CARS-XGBoost configuration produced a lower test accuracy of 91.45%. For SVM, the SG-SPA combination produced a test accuracy of 96.23% and a training accuracy of 96.11%. SVM paired with SG-LARS and SG-CARS yielded accuracy values within a similar range. PLS-DA and RF produced lower test accuracies, with the highest PLS-DA accuracy below 88% and RF around 80%. Consistently, their average ranks were also above 8.0 (all above 8.0, with SG-CARS-RF reaching 8.99).

**Table 3:** Classification models based on different characteristic bands selection approaches.

Feature selection	Model	Dataset type	Accuracy	Precision	Recall	F1-score	CV accuracy	Avg. rank
SG-SPA	ANN	Train	99.33%	99.33%	99.33%	99.33%	99.20 ± 0.14%	7.34
		Test	98.88%	98.88%	98.88%	98.88%		
	XGBoost	Train	97.81%	97.85%	97.81%	97.81%	97.57± 0.29%	7.45
		Test	97.01%	97.04%	97.01%	97.01%		
	SVM	Train	96.11%	96.13%	96.11%	96.11%	96.15 ± 0.32%	7.60
		Test	96.23%	96.24%	96.23%	96.23%		
	PLS_DA	Train	87.40%	87.22%	87.40%	87.26%	87.31 ± 0.73%	8.09
		Test	87.09%	86.86%	87.09%	86.94%		
	RF	Train	81.51%	82.61%	81.51%	81.31%	81.19 ± 0.20%	8.54
		Test	80.44%	81.49%	80.44%	80.24%		
SG-LARS	ANN	Train	98.82%	98.83%	98.82%	98.82%	98.75 ± 0.19%	7.38
		Test	98.60%	98.61%	98.60%	98.60%		
	XGBoost	Train	97.13%	97.18%	97.13%	97.13%	96.82 ± 0.45%	7.57
		Test	96.08%	96.13%	96.08%	96.08%		
	SVM	Train	95.78%	95.78%	95.78%	95.77%	95.78 ± 0.17%	7.59
		Test	95.79%	95.81%	95.79%	95.78%		
	PLS_DA	Train	79.90%	79.68%	79.90%	79.69%	79.65 ± 0.59%	8.85
		Test	79.05%	78.82%	79.05%	78.85%		
	RF	Train	80.58%	82.17%	80.58%	80.24%	80.31 ± 0.59%	8.80
		Test	79.67%	81.12%	79.67%	79.32%		
SG-CARS	ANN	Train	97.80%	97.80%	97.80%	97.80%	97.63 ± 0.46%	7.49
		Test	97.24%	97.24%	97.24%	97.24%		
	XGBoost	Train	92.42%	92.53%	92.42%	92.40%	92.13 ± 0.40%	7.92
		Test	91.45%	91.49%	91.45%	91.43%		
	SVM	Train	91.43%	91.41%	91.43%	91.42%	91.54 ± 0. 55%	7.92
		Test	91.79%	91.77%	91.79%	91.77%		
	PLS_DA	Train	84.91%	84.77%	84.91%	84.82%	85.00 ± 0.18%	8.45
		Test	85.20%	85.07%	85.20%	85.10%		
	RF	Train	80.51%	80.99%	80.51%	80.38%	80.37 ± 1.69%	8.99
		Test	80.03%	80.56%	80.03%	79.93%		

The Friedman test revealed significant differences among models (χ ² = 9347.20, p< 0.0001). Average ranks are computed based on sample-level predictions.

**Figure 9:**
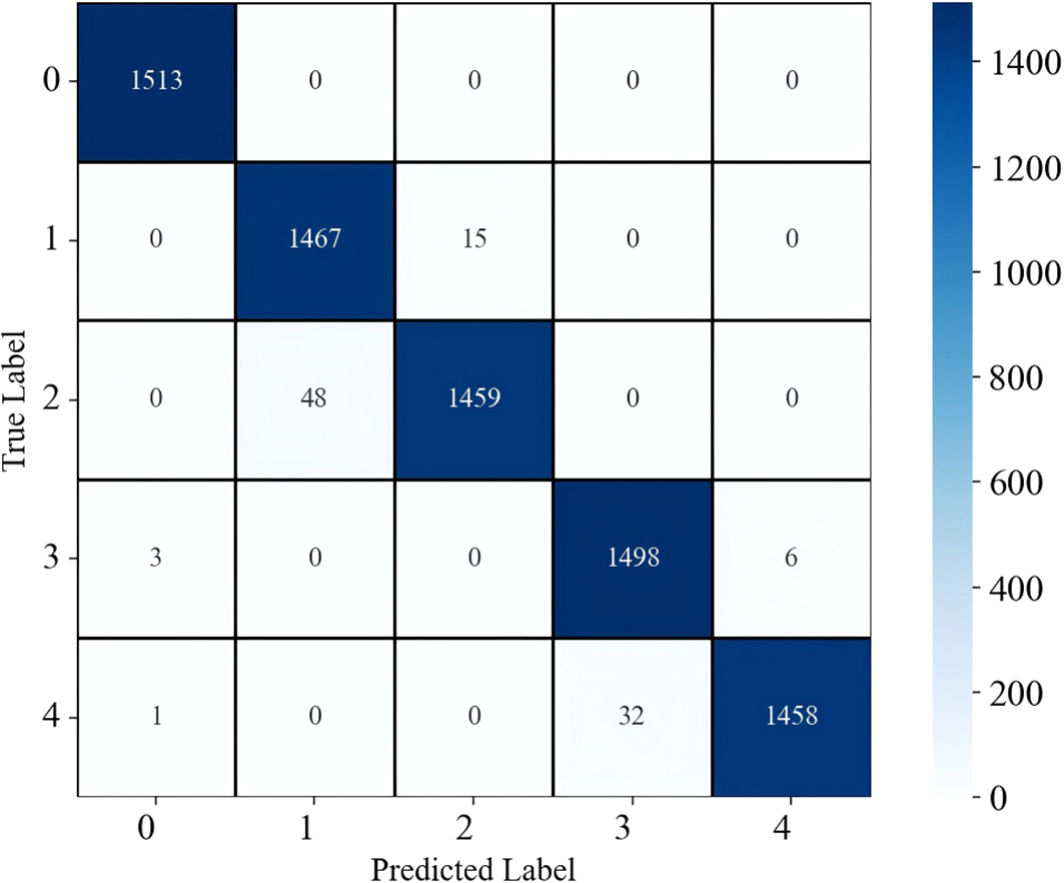
Confusion matrix of the SG-SPA-ANN model on the test set. Labels 0, 1, 2, 3, and 4 correspond to healthy, brown spot, wildfire, TMV, and PVY, respectively.

Compared with full-band models in **Table 2**, feature-band classification models reduced input dimensionality with similar accuracy. Although the test accuracy of full-band SG-ANN reached 98.93%, the test accuracy of feature-band SG-SPA-ANN was 98.88%, while input dimensionality decreased from all wavelengths to 20 representative bands. Under SG-SPA, this comparison shows that feature-band models produced smaller differences between training and test accuracy than some full-band models. **Figure 9** shows the confusion matrix for SG-SPA-ANN on the test set. **Table 4** summarizes the multi-class evaluation metrics, and **Table 5** details the per-class results. The model reached an overall accuracy of 98.88%. All 1,510 samples of healthy leaves were correctly identified; 1,457 samples of brown spot and 1,487 wildfire were correctly classified, with accuracies each exceeding 98.3%; the accuracies of TMV and PVY reached 99.07% and 98.53%, respectively. Misclassifications occurred primarily between lesion-type and virus-type classes.

**Table 4:** Classification performance of the SG-SPA-ANN model for tobacco leaf disease detection.

Class	Precision	Recall	F1-score
Healthy Tobacco	99.93%	99.80%	99.86%
Tobacco Brown Spot	98.65%	98.31%	98.48%
Tobacco Wildfire	98.35%	98.68%	98.51%
Tobacco Mosaic Virus	98.61%	99.07%	98.84%
Potato virus Y	98.86%	98.53%	98.69%
Average	98.88%	98.88%	98.88%

**Table 5:** Performance comparison of SG-SPA-ANN and X-Transformer models on tobacco disease classification.

Model	Input features	Train accuracy	Test accuracy	Gap	Precision	Recall	F1-score
SG-SPA-ANN	20	99.33%	98.88%	0.45%	98.88%	98.88%	98.88%
X-Transformer	462	100.00%	99.35%	0.65%	99.35%	99.35%	99.35%

##### 3.4.2.2 Performance of the X-transformer model

**Figure 10:**
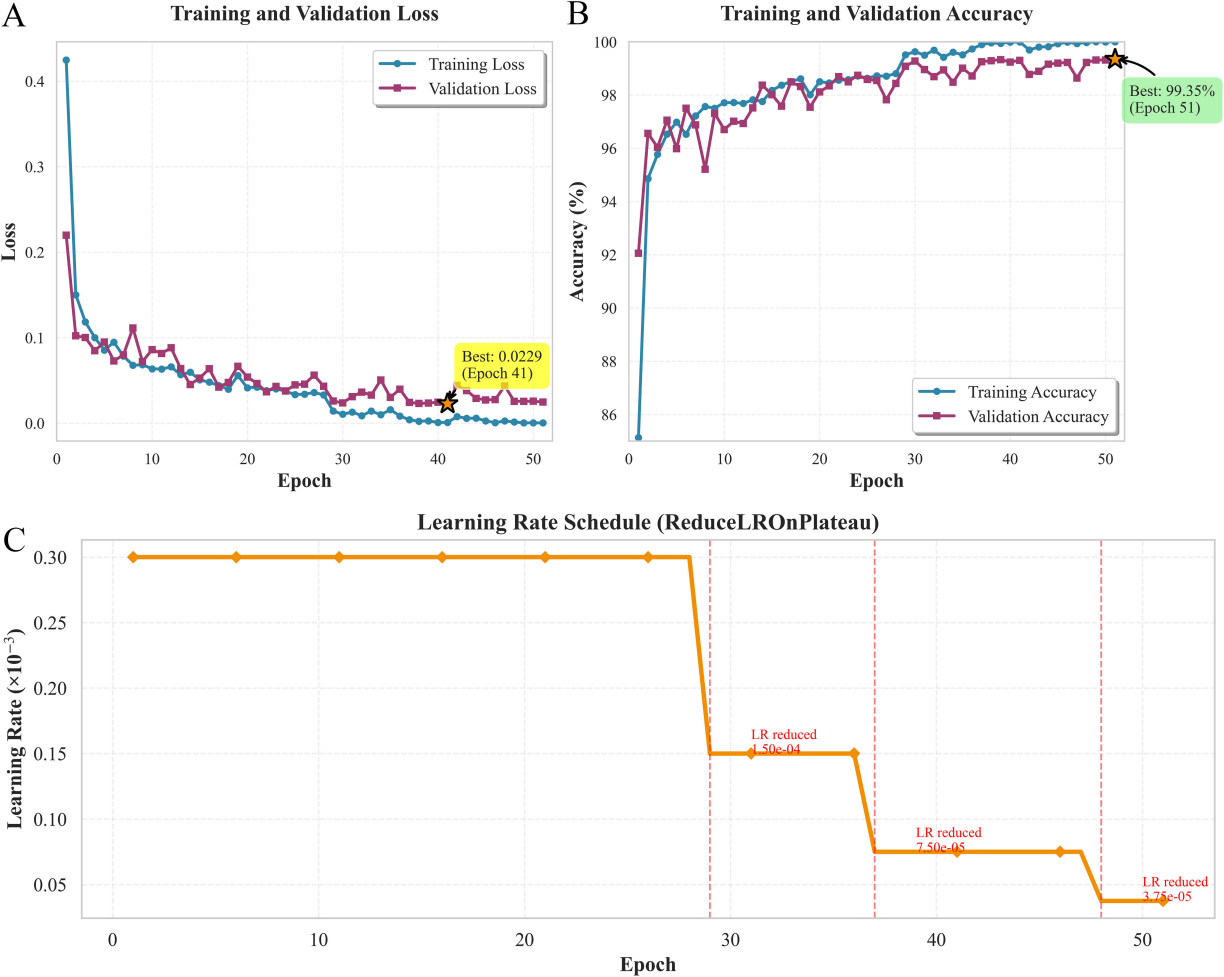
Visualization of the X-Transformer model training process. **(A)** Training and validation loss curves; **(B)** Training and validation accuracy curves; **(C)** Adaptive adjustment of the learning rate over training epochs (ReduceLROnPlateau strategy).

The X-Transformer model exhibited stable learning dynamics and strong generalization capability during training. As shown in the loss curve (**Figure 10A**), the training loss decreased steadily during the 51 epochs of training from an initial value of 0.425 to 0.00025. During training, the validation loss reached its minimum value of 0.0229 at epoch 41 and then stabilized at around 0.025. Therefore, the model trained until epoch 51 had essentially converged, and we did not observe any sign of overfitting at the end of training. The validation accuracy curve (**Figure 10B**) supported this conclusion: training accuracy steadily increased during training to 100%, and validation accuracy plateaued at 99.35%. The small difference between the two suggests that the model had a good generalization capability. To avoid potential degradation during the later training process, we used an early-stopping method. When monitoring the validation loss showed no improvement for 10 consecutive epochs, training was automatically stopped at epoch 51, and the best model weights corresponding to the epoch with the best validation performance were saved (at epoch 41). The learning rate was scheduled using the ReduceLROnPlateau strategy (**Figure 10C**) with an initial rate of 3 × 10^-4^. When the validation loss did not decrease for five consecutive epochs, the learning rate was reduced to half of its previous value (at epochs 29, 37, and 48), resulting in a learning rate of 3.75 × 10^-5^.

##### 3.4.2.3 Comparison between SG-SPA-ANN and X-transformer

To further evaluate the effectiveness of the proposed traditional ML framework, a deep learning method based on X-Transformer model was added to the comparison. X-Transformer uses multi-head self-attention to model long-term dependencies among different spectral features in an end-to-end manner, and has become one of the most popular DL methods for hyperspectral classification in recent years. In contrast, the proposed SG-SPA-ANN model was developed based on SG smoothing and SPA-based feature selection with ANN classifier, allowing it to retain interpretability while reducing feature dimensionality. The comparative results are shown in **Table 5**. SG-SPA-ANN achieved a 0.45% training-test accuracy gap, while X-Transformer showed a slightly larger gap of 0.65%. Although the X-Transformer achieved slightly higher numerical accuracy, the gap between them was only 0.47%. In addition, SG-SPA-ANN showed better training-validation consistency. Therefore, the traditional ML approach remains competitive for hyperspectral data classification. In particular, SG-SPA-ANN model demonstrated better stability, higher generalization ability when the sample size was limited and data dimensionality was high. Additionally, compared with other deep learning models, the SG-SPA-ANN model offers a compact architecture, higher interpretability. Furthermore, the trained model was still able to achieve high classification accuracy within the feature space, indicating that the model was applicable to low-dimensional spectral sensing or even single-band imaging system. In the future, we plan to select the most discriminative spectral bands for designing lightweight optical lenses or embedded agricultural monitoring devices targeting specific wavelengths, to achieve low-cost and high-efficiency disease identification.

In addition to classification accuracy, the two models differ in model size and input dimensionality. X-Transformer uses all 462 spectral bands and contains approximately 4.84 million trainable parameters. In contrast, SG-SPA-ANN takes only 20 SPA-selected wavelengths as input and uses a shallow ANN architecture. These design choices reduce the number of input features and parameters for SG-SPA-ANN compared with X-Transformer. This difference in complexity indicates a potential reduction in computational cost for SG-SPA-ANN, although detailed timing measurements were beyond the scope of the present study and are not reported here.

### 3.5 SHAP interpretation of the SG-SPA-ANN model

**Figure 11:**
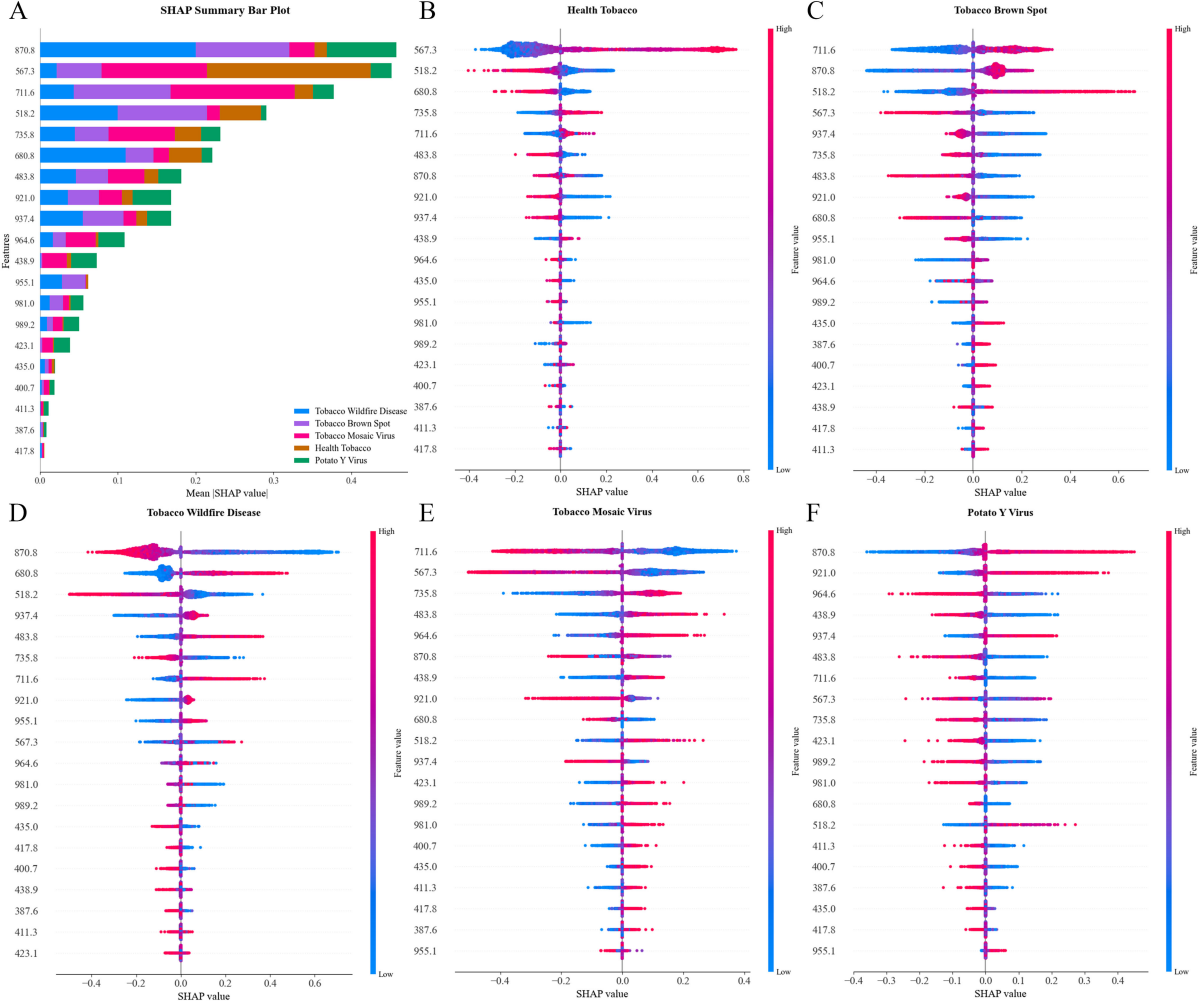
Interpreting the model based on SHAP **(A)** SHAP feature importance bar plot, depicting the mean absolute SHAP values of 20 characteristic bands as indicators of their overall importance in the model. The horizontal axis represented the mean absolute SHAP values, while the vertical axis listed the 20 characteristic bands, arranged in descending order of importance. **(B–F)** Bee swarm plots, illustrating the SHAP values for different categories. **(B)** healthy, **(C)** brown spot, **(D)** wildfire, **(E)** TMV, and **(F)** PVY.

In order to explain the basis of the SG-SPA-ANN classification model for distinguishing five tobacco (four disease types and one healthy), this study conducted an interpretability analysis based on SHAP. As shown in **Figure 11A**, there were significant differences in the effects of different features on the model output. Among them, the SHAP value at 870.8 nm was much higher than that of other features, making it the most critical factor driving the model prediction. In addition, spectral bands such as 567.3 nm and 711.6 nm also contributed substantially to the model’s decisions. There were significant differences in the most critical discriminative features used by different disease types. Specifically, 870.8 nm was particularly important for identifying wildfire, brown spot, and PVY; the feature at 567.3 nm contributed prominently to distinguishing healthy tobacco; and 711.6 nm contributed greatly to differentiating between brown spot and TMV.

The bee swarm plot illustrates the global importance and directional influence of spectral features in diagnosing various disease categories (**Figures 11B–F**). For healthy tobacco, the key feature was the reflectance value at 567.3 nm. Low values reduced the probability of the model classifying it as healthy through negative SHAP effects, while high values increased this tendency via positive SHAP effects. Healthy tobacco exhibits high and stable reflectance in the green light region due to vigorous chlorophyll synthesis. Disease-induced chlorophyll degradation and cellular structural damage caused reduced reflectance in this band. Key discriminative features for brown spot were concentrated at 711.6 nm (red-edge region) and 870.8 nm (near-infrared region). High SHAP values at 711.6 nm reflected enhanced red-edge reflectance associated with mesophyll cell damage, whereas low SHAP values at 870.8 nm helped reduce confusion with wildfire through inhibitory effects. Reduced near-infrared reflectance in leaves infected with brown spot and wildfire likely resulted from pathogen-induced degradation of mesophyll cell structure and collapse of intercellular air spaces. This diminishes the number of internal scattering centers and weakens near-infrared reflectance. Key features for wildfire included low values at 870.8 nm (decreased NIR reflectance due to cell necrosis) and high values at 680.8 nm (increased scattering from disrupted cell structure), jointly forming its spectral identification basis. TMV detection relied on abnormal responses at 711.6 nm (reduced red edge reflectance) and 567.3 nm (decreased green light reflectance), corresponding to chlorosis and dehydration pathological manifestations, respectively. Key contributing features for PVY were enhanced near-infrared reflectance at 870.8 nm and 921.0 nm, associated with cell structure damage due to dehydration and vascular cell wall disruption, respectively. SHAP analysis elucidated the underlying mechanisms by linking spectral-band behavior with SHAP value patterns: stable chlorophyll reflectance in healthy tobacco, red-edge disturbances in brown spot, enhanced NIR scattering in wildfire, and chlorosis- or cell-damage-related signatures in viral infections. These findings validate the effectiveness of the model and provide an interpretable scientific basis for hyperspectral diagnosis of tobacco diseases.

### 3.6 Visualization and severity grading of brown spot and wildfire

**Figure 12:**
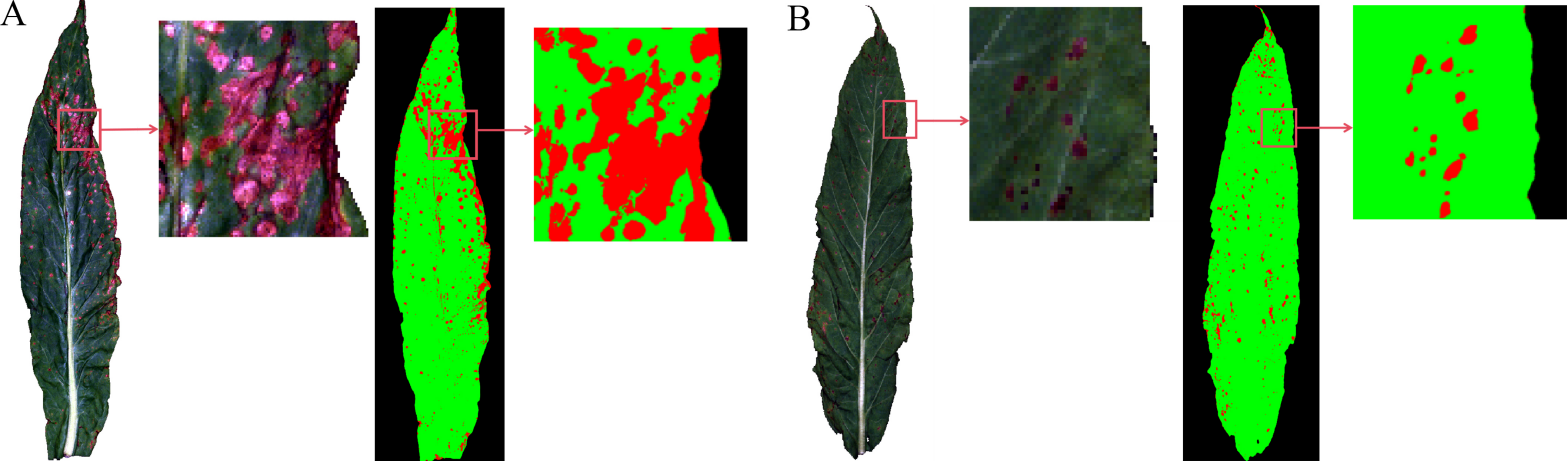
Visualization of damage caused by brown spot and wildfire. **(A)** A pseudo-color composite image of a tobacco brown spot leaf (left) and the model prediction result of pixel-level classification using the optimal SG-SPA-ANN model (right). The lesion area proportion was 13.86%, and the severity level was 7; **(B)** A pseudo-color composite image of a wildfire leaf (left) and the corresponding model prediction result (right). The lesion area proportion was 3.71%, and the severity level was 3. The figure indicated by the arrow is an enlarged view within the red box.

To evaluate the performance of the model in the spatial dimension, the optimal SG-SPA-ANN classifier was applied to pixel-level prediction on hyperspectral images of brown spot and wildfire leaves. The classification results were visualized through pixel-wise prediction combined with pseudo-color mapping, to show the spatial distribution of healthy tissues and lesion areas. As shown in **Figure 12**, the model produced lesion maps that closely aligned with visual inspection, with diseased regions highlighted in red and healthy tissues in green. Distinct spatial patterns were observed between predicted lesion regions and predicted healthy regions.

**Figure 13:**
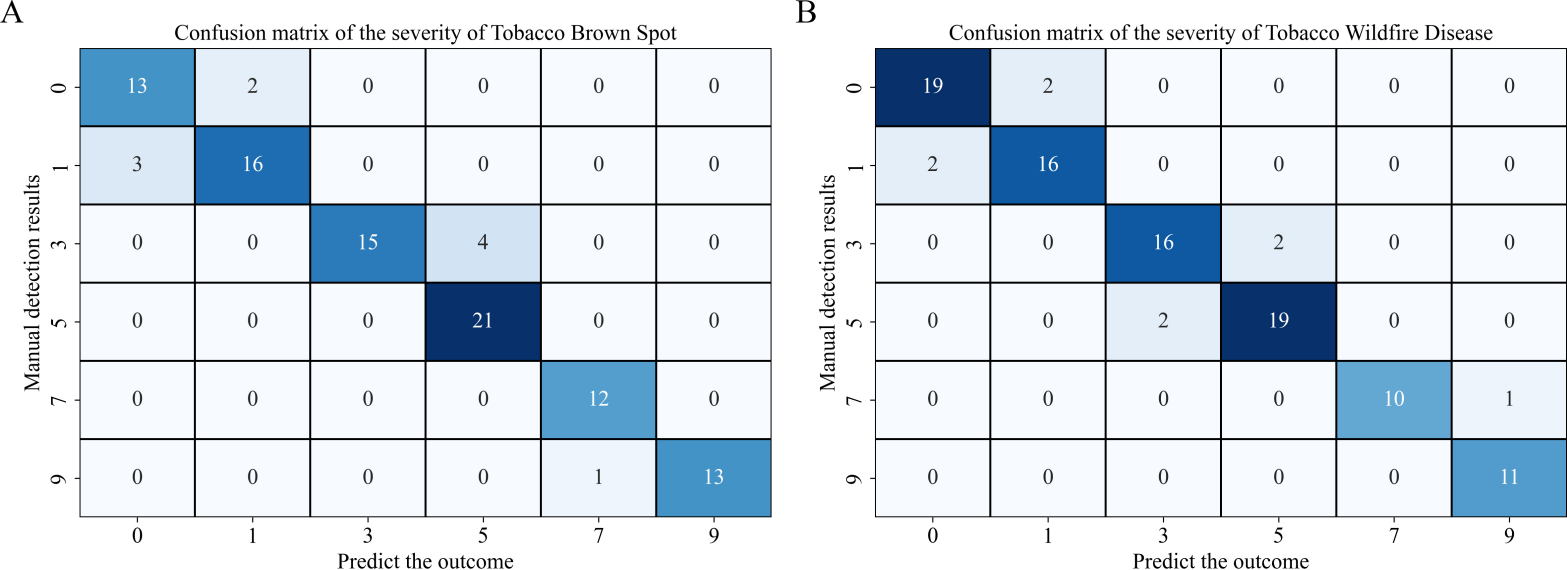
Confusion matrices of severity grading. **(A)** brown spot; **(B)** wildfire.

Through confusion matrix analysis of severity grading **Figure 13** most severity levels were correctly distinguished by the pixel-based lesion segmentation combined with lesion-area statistics. The main diagonal elements showed concentrated values, indicating strong agreement with manual labels. Specifically, brown spot levels 0, 5, 7, and 9 showed high accuracy, and were correctly classified, with some confusion occurring between levels 3 and 5, which was expected because adjacent levels share similar spectral patterns. Similarly, most wildfire samples were correctly assigned, with occasional misclassification between neighboring levels. Overall, both diseases achieved severity-classification accuracies above 90% on the independent test set, with misclassifications mainly occurring between adjacent severity levels.

## 4 Discussion

This study assessed the effectiveness of a compact hyperspectral framework for identifying five major tobacco leaf conditions. By combining Savitzky-Golay preprocessing, SPA-based wavelength selection, and a lightweight ANN classifier, the framework achieved stable multi-class performance using only 20 selected wavelengths. These findings show that accurate multi-disease discrimination can be achieved without relying on full-spectrum data or large deep-learning models. Pixel-level predictions further enabled estimation of lesion distribution and severity for brown spot and wildfire, suggesting that the framework can support quantitative disease assessment.

SHAP analysis helped clarify how the selected wavelengths contributed to classification. The most influential bands-567.3 nm, 711.6 nm, and 870.8 nm-correspond to spectral regions linked to pigment changes, red-edge shifts, and reduced near-infrared scattering. These patterns are consistent with earlier hyperspectral studies that reported chlorophyll loss in viral diseases (Zhu et al., 2017), disturbances in pigment concentration and mesophyll structure (Sims and Gamon, 2002; Chen et al., 2023), and reduced internal scattering caused by fungal infections (Gu et al., 2019). The agreement between SHAP-derived patterns and known physiological responses suggests that the model relied on meaningful spectral cues. However, because no physiological measurements were collected alongside spectral data, these interpretations remain inferential.

This study extends previous hyperspectral research on tobacco diseases in several key ways. Earlier work focused mainly on binary classification or single viral diseases such as TMV or PVY (Zhu et al., 2017; Chen et al., 2023; Pan et al., 2023). These studies demonstrated the promise of hyperspectral imaging but did not address fungal diseases or multi-class scenarios. By integrating both fungal and viral pathogens into a unified five-class model, the present study expands the diagnostic scope and shows that hyperspectral imaging retains strong discriminative ability across more diverse disease types. The use of only 20 SPA-selected wavelengths further reduces spectral dimensionality by over 95% compared with full-spectrum models used in prior work. This reduction provides a practical foundation for designing lightweight multispectral or narrowband sensors. The ability to map lesions at the pixel level also highlights the potential application of the framework in supporting severity analysis, rather than simply classifying disease type. From a computational perspective, using only 20 bands and a shallow ANN also reduces model size compared with the 4.84M-parameter X-Transformer baseline. This compact design may facilitate deployment on devices with limited memory and processing resources, although runtime differences were not quantified in this study.

Despite these strengths, several limitations should be considered. All hyperspectral images were acquired under controlled indoor illumination, which differs from natural field conditions where light, background, and leaf geometry vary substantially. The number of independent leaf samples was limited, especially for viral diseases, and mild symptoms were underrepresented. This imbalance may reduce sensitivity to early-stage infections. Reduced performance observed when testing spectra from different cultivars suggests that cross-cultivar generalization requires further investigation. In addition, although SHAP analysis identified physiologically meaningful wavelengths, no biochemical or structural measurements were collected to confirm these interpretations.

Future studies should expand sample diversity by including more cultivars, symptom stages, and environmental conditions. Integrating physiological measurements, such as chlorophyll content or tissue microstructure, would help validate the mechanisms underlying the SHAP patterns. Testing the workflow under natural field conditions will be essential for assessing robustness in operational settings. Finally, techniques such as transfer learning or domain adaptation may help improve generalization across production regions and environmental conditions.

## 5 Conclusions

This study implemented an SG-SPA-ANN framework to examine the use of hyperspectral imaging for identifying five tobacco leaf conditions. The combination of Savitzky-Golay preprocessing, SPA-based wavelength selection, and an artificial neural network classifier enabled multi-class discrimination and supported pixel-level lesion mapping for brown spot and wildfire. Using a small set of selected wavelengths, the model achieved high accuracy under controlled conditions while requiring fewer computational resources than a full-spectrum Transformer benchmark. SHAP analysis showed that several influential wavelengths corresponded to spectral regions commonly associated with pigment- and structure-related changes. These findings indicate that wavelength-reduced models may provide a practical option for tobacco disease identification in controlled imaging environments. Further evaluation across cultivars, disease stages, and field conditions will be necessary to assess the general applicability and robustness of the approach.

## Data Availability

The original contributions presented in the study are included in the article/supplementary material. Further inquiries can be directed to the corresponding authors.
